# Multi-Axle Reference and Temporal-Consistency Deep SVDD for EMU Traction Motor Bearing Anomaly Detection Using Field Vibration Data

**DOI:** 10.3390/s26154891

**Published:** 2026-08-03

**Authors:** Qi Wu, Xiaomin Zhu, Zhikai Jia, Zhongkai Wang

**Affiliations:** 1 Institute of Computing Technology, China Academy of Railway Sciences Corporation Limited, Beijing 100081, China; qiiiwuuu@163.com (Q.W.); jzkemail@163.com (Z.J.); 2 School of Mechanical, Electronic and Control Engineering, Beijing Jiaotong University, Beijing 100044, China; xmzhu@bjtu.edu.cn

**Keywords:** EMU traction motor bearing, anomaly detection, field vibration monitoring, multi-axle reference, Deep SVDD

## Abstract

Field vibration monitoring of EMU traction motor bearings is commonly constrained by weak or relative labels, fluctuations in operating conditions, and limited abnormal samples. Under these conditions, learning a normal boundary from a single bearing position may be unstable, and isolated score spikes may lead to unreliable alarms. To address these issues, this study proposes a multi-axle reference and temporal-consistency-enhanced Deep SVDD framework, termed MA-TC-Deep SVDD, for field anomaly detection of EMU traction motor bearings. Unlike closed-set fault diagnosis that requires known fault labels, the proposed framework focuses on identifying deviations from the stable operating regime. First, a compact 10-dimensional time-frequency representation is constructed from valid vibration segments. Second, stable samples from the target bearing position and screened stable samples from other monitored positions on the same EMU are organized as a multi-axle reference set for one-class normal-boundary learning. Third, feature recalibration, temporal-consistency regularization, reference-score standardization, causal smoothing, and consecutive-alarm judgment are incorporated to improve robustness against field disturbances. The anomaly-prior-guided health-state interpretation module is retained only as post hoc evidence for describing severity evolution and does not feed back into the anomaly detection threshold. Field data collected from an in-service EMU over D1–D5 are used for validation. The results show that bearing position 1 has low anomaly scores on D1–D2, exhibits transitional deviation on D3, and shows persistent state deviation on D4–D5, while the other monitored positions remain comparatively stable. Under the current weak-label evaluation protocol, MA-TC-Deep SVDD achieves higher average anomaly detection performance than the compared baseline methods, with AUC = 0.909, AP = 0.872, Precision = 0.887, Recall = 0.802, F1 = 0.843, and FAR = 0.047. These results indicate that the proposed framework can provide field anomaly-warning and severity-oriented interpretation under weak-label monitoring conditions. However, it should not be interpreted as a replacement for disassembly-confirmed fault-type diagnosis or remaining useful life prediction.

## 1. Introduction

EMU traction motor bearings are key components of the traction system, and their vibration behavior is closely related to operational safety and maintenance reliability [1]. Compared with laboratory datasets, field monitoring data are more difficult to model because abnormal labels are scarce, working conditions fluctuate, and segment quality is not fully controllable [2]. Therefore, anomaly detection based mainly on stable operating data is more suitable than closed-set fault classification when the abnormal state is unknown or only weakly labeled. Classical rolling-bearing vibration knowledge still provides the physical basis for feature interpretation [3], while support vector data description and deep one-class learning provide a feasible modeling route for learning compact normal-state boundaries [4,5]. Existing unsupervised and self-supervised bearing studies have shown that abnormal deviations can be detected without complete fault-type labels [6,7,8]. More recently, deep sequential models have been increasingly used for industrial time-series anomaly detection. Recurrent autoencoders, temporal convolutional networks, and Transformer-based models can capture temporal dependencies and long-range feature interactions in vibration or multi-sensor signals. In addition, self-supervised representation learning and contrastive learning have been explored to reduce the dependence on complete fault labels. Large language models and foundation models have also attracted attention in intelligent maintenance, mainly for maintenance knowledge reasoning, diagnostic report generation, and human–machine interaction. However, their direct application to high-frequency bearing vibration anomaly detection remains limited, because reliable signal-level representation learning and field-data validation are still required. Therefore, for weakly labeled EMU traction motor bearing monitoring, a compact and physically interpretable anomaly detection framework remains practically necessary.

Although recent sequential and representation learning methods improve temporal feature extraction, they do not directly resolve two practical issues considered in this study: how to construct a reliable normal reference when fault labels are unavailable, and how to suppress isolated alarm responses caused by field operating fluctuations. Therefore, the present study focuses on weak-label reference-set construction and temporally consistent one-class decision-making rather than closed-set fault classification.

Recent studies have further improved bearing anomaly detection through dual-input networks, autoencoder-WGAN models, VMD-assisted learning, and optimized VMD with deep temporal models [9,10,11,12]. In railway applications, wheelset-bearing sparse-coding detection and high-speed-train axle-bearing time-frequency analysis have also demonstrated the value of vibration-based monitoring under complex operating conditions [13,14]. These studies provide useful references, but many of them still rely on either laboratory data, predefined fault categories, or prediction-error pipelines. For EMU traction motor bearings in field service, the detector must additionally distinguish local bearing-position deviation from train-level operating fluctuation.

Online anomaly detection and cross-machine diagnosis indicate that practical railway deployment requires continuous warning ability and robustness to machine-to-machine variability [15,16]. A recent EMU motor-bearing study based on VMD and deep learning is close to the present application scenario, but it identifies abnormality through prediction residuals and OC-SVM rather than learning a Deep SVDD normal boundary from multi-position stable references [17]. Accordingly, the present study is organized around three field difficulties: insufficient reliable abnormal labels, unstable single-position normal boundaries, and isolated score fluctuations caused by transient operating disturbances.

This study proposes an anomaly detection method for field vibration signals of EMU traction motor bearings. The proposed MA-TC-Deep SVDD framework follows the practical logic of field monitoring. The raw vibration records are first divided into valid monitoring segments, and a compact set of time-frequency descriptors is extracted to characterize vibration energy, fluctuation, impulsiveness, impact behavior, spectral distribution, and dominant-frequency information. Stable samples from the target bearing position, screened stable fragments from the transitional day, and stable samples from other monitored positions are then organized as a multi-axle reference set. This reference set is used to train a Deep SVDD detector that learns a compact normal boundary in the latent feature space. Feature recalibration, temporal-consistency regularization, reference-score standardization, causal smoothing, and consecutive-alarm judgment are further combined to transform segment-level distance scores into reliable anomaly warnings. Finally, APG-TA-SAE-DMHI is retained only as an auxiliary post hoc health-state interpretation layer to describe severity evolution after the anomaly decision has been obtained. It does not participate in the primary anomaly thresholding process. Therefore, the methodological focus of this study is not to replace fault-type diagnosis, but to provide a weak-label field anomaly-warning workflow with physically interpretable evidence.

Unlike previous studies that rely mainly on laboratory datasets, predefined fault categories, or prediction residuals followed by a conventional one-class classifier, this framework is validated on field monitoring data and uses same-train cross-position stable evidence to define the normal regime. This design is consistent with the practical situation of EMU traction motor bearing monitoring, where abnormal samples are limited, operating conditions fluctuate, and the alarm result must distinguish localized bearing-position deviation from train-level common disturbance.

The functional roles and synergistic interactions of the proposed components are summarized as follows:

- The compact time-frequency representation functions as the field-data interface. It converts long vibration records into segment-level descriptors with clear physical meaning, so that the detector can use energy-, fluctuation-, impulsiveness-, impact-, and spectrum-related evidence without depending on a high-dimensional raw-signal input.
- The multi-axle reference-guided Deep SVDD model operates as the core one-class boundary learner. By combining stable samples from the target bearing position with synchronous stable samples from other monitored positions, it expands the reference distribution and reduces the boundary bias that may occur when only a single bearing position is used for normal-state modeling.
- The temporal-consistency and engineering alarm module converts latent-space distance into deployable warning information. Temporal consistency constrains adjacent monitoring segments, reference-score standardization makes distance values comparable, causal smoothing suppresses isolated spikes, and consecutive exceedance judgment prevents transient disturbances from being directly reported as persistent anomalies.
- The APG-TA-SAE-DMHI module serves as a post hoc severity interpreter rather than a second detector. Anomaly-prior weighting reduces the influence of suspicious samples on the health reference, temporal attention encodes short-window continuity, and dynamic Mahalanobis distance produces a bounded health indicator for explaining cross-day state evolution.

This integrated framework establishes a coherent workflow tailored to weak-label field monitoring of EMU traction motor bearings. The novelty of the proposed framework lies mainly in adapting and integrating multi-axle reference learning, temporal-consistency-constrained Deep SVDD, and post hoc health-state interpretation for weakly labeled field vibration monitoring. The main contributions of this study are as follows:

- A compact and physically interpretable feature interface is constructed for weak-label field vibration monitoring. Ten time-frequency descriptors are used to represent each monitoring segment, preserving bearing-vibration evidence related to energy, fluctuation, impulsiveness, impact level, spectral distribution, and dominant-frequency behavior while reducing the risk of overfitting under limited abnormal labels.
- A multi-axle reference-guided one-class boundary learning strategy is developed for field anomaly warning. Stable samples from the target bearing position and screened stable samples from other monitored positions are jointly used as same-train reference evidence, so that Deep SVDD can learn a more robust normal boundary under operating-condition variability.
- A temporal-consistency-constrained alarm strategy is introduced to reduce false reactions to isolated field disturbances. Feature recalibration, temporal consistency, reference-score standardization, causal smoothing, and consecutive-alarm judgment are integrated to emphasize persistent state deviation rather than single-segment score spikes.
- An auxiliary post hoc health-state interpretation strategy is retained to describe the severity evolution of detected cross-day deviation. APG-TA-SAE-DMHI provides maintenance-oriented interpretation after the anomaly score has been obtained, while the primary decision logic remains centered on MA-TC-Deep SVDD.

## 2. Methodology

The methodology is organized according to the actual deployment logic of field monitoring. Section 2.1 describes how raw vibration records are converted into compact feature samples and mutually independent data subsets. Section 2.2 presents multi-axle reference construction and Deep SVDD boundary learning. Section 2.3 further explains temporal-consistency regularization and the engineering alarm decision. Section 2.4 introduces the post hoc APG-TA-SAE-DMHI interpretation module, and Section 2.5 defines the evaluation protocol.

### 2.1. Field Data Organization and Compact Time-Frequency Representation

This subsection defines how field vibration records are converted into samples for one-class anomaly detection. The field case uses vibration records collected from traction motor bearings of an in-service EMU. For clarity, D1–D5 denote five chronological monitoring dates/days rather than bearing or axle positions. Bearing positions 1–4 denote four monitored traction-motor bearing locations on the same EMU. Bearing position 1 is selected as the target position for anomaly evaluation, while bearing positions 2–4 are used only as same-train stable reference/control positions.

The vibration records were acquired from traction motor bearings of an in-service EMU through an engineering monitoring system. The sampling frequency was fixed at 10 kHz. The monitored records cover start-up, acceleration, approximately steady-speed operation, deceleration, and stopping. Therefore, the bearing was not monitored under a constant-speed or constant-load condition. The measured vibration responses contain the combined influence of speed variation, load fluctuation, line excitation, structural transmission-path variation, and environmental disturbance. Owing to confidentiality requirements associated with the railway engineering dataset, the exact vehicle and route identifiers, detailed sensor specifications, mounting configuration, operating ranges, and service-run metadata are not disclosed. The signal-processing, sample-partition, and model-implementation settings used in this study are reported below.

The term “bearing position” is used consistently in the following sections and figures. Unless the physical axle number is explicitly discussed, the term “axle” is avoided to prevent confusion between monitoring dates D1–D5 and bearing positions 1–4.

The input of the detector is designed as a compact segment-level feature vector rather than a raw long sequence. This choice is based on two considerations. First, the field dataset contains weak labels and uneven operating states, so a directly trained high-dimensional raw-signal network may easily learn condition fluctuation rather than bearing-state deviation. Second, vibration features can retain engineering interpretability and make the subsequent one-class boundary easier to analyze. Therefore, each valid segment is represented by ten time-frequency descriptors, including four time-domain features related to vibration energy, global fluctuation, impulsiveness, and impact level, together with six frequency-domain features describing middle- and high-frequency energy distribution, spectral centroid, spectral spread, spectral entropy, and dominant-frequency information. Stable fragments on D3 were screened before model training using only reference-side statistical stability criteria. The screening procedure did not use D4–D5 samples or the final anomaly detection results. Candidate D3 segments were retained as stable fragments only when their feature amplitudes, local variance, impulsiveness-related indicators, and spectral-energy descriptors remained within the stable range estimated from D1–D2 and the stable reference positions. Segments with abrupt amplitude changes, abnormal impulsiveness, incomplete acquisition, or strong deviation from the reference feature range were excluded from the reference pool and retained only for transition evaluation. This procedure was used to reduce the risk of including hidden abnormal samples in the reference set.

The sample construction procedure contains four steps. Step 1 first partitions the original continuous vibration records according to monitoring date/run, so that records assigned to reference training, reference validation, transition evaluation, main-deviation evaluation, and stable-control subsets are separated before segment-level processing. Step 2 divides each continuous record into fixed-length 1 s monitoring segments. At the sampling frequency of 10 kHz, each segment contains 10,000 sampling points. Before frequency-domain feature extraction, each segment is demeaned and windowed, followed by a fast Fourier transform. With *f*_s_ = 10,000 Hz and *N* = 10,000, the frequency resolution is 1 Hz, and the one-sided spectrum contains 5001 frequency points covering the range from 0 to 5 kHz. Step 3 removes invalid segments caused by incomplete acquisition, signal interruption, or identifiable non-operating states. Step 4 extracts the ten-dimensional time-frequency feature vector from each valid segment and organizes the samples into reference-training, reference-validation, transition-evaluation, main-deviation-evaluation, and stable-control subsets. The reference pool includes stable samples from bearing position 1 on D1–D2, screened stable fragments from bearing position 1 on D3, and stable samples from bearing positions 2–4. The stable-control set is not used for model training, threshold determination, or score standardization, so that it can serve as an independent false-alarm verification set.

After feature extraction and sample partitioning, features with different physical scales must be standardized before one-class boundary learning. Each vibration segment is represented as a 10-dimensional feature vector:

(1) $$x_{i}={\left[x_{i1},x_{i2},\ldots ,x_{i10}\right]}^{T}$$

where *x_i_* denotes the feature vector of the *i*-th segment, and *x_ij_* is the *j*-th feature. Feature standardization is calculated as

(2) $${\hat{x}}_{ij}=\frac{x_{ij}-\mu_{j}}{\sigma_{j}+\varepsilon }$$

where *μ_j_* and *σ_j_* are the mean and standard deviation of the *j*-th feature calculated from the reference-training set, and *ε* is a small constant used to avoid division by zero.

To avoid information leakage caused by overlapping sliding windows, windows generated from the same original run/day segment were not assigned to different subsets. Therefore, adjacent or overlapping windows from the same continuous vibration record do not appear simultaneously in training and evaluation subsets.

To avoid information leakage, data partitioning was performed at the original continuous-record and monitoring-day level before segment allocation. Segments originating from the same continuous record were not assigned to different training and evaluation subsets. In addition, the transition-evaluation, main-deviation-evaluation, and stable-control samples were not used for network training, hyperparameter selection, alarm-threshold determination, or reference-score standardization. This partitioning strategy reduces the risk that temporally adjacent samples from the same source record appear simultaneously in the training and evaluation subsets. The resulting sample partition and the role of each subset are summarized in Table 1.

**Figure 1:**
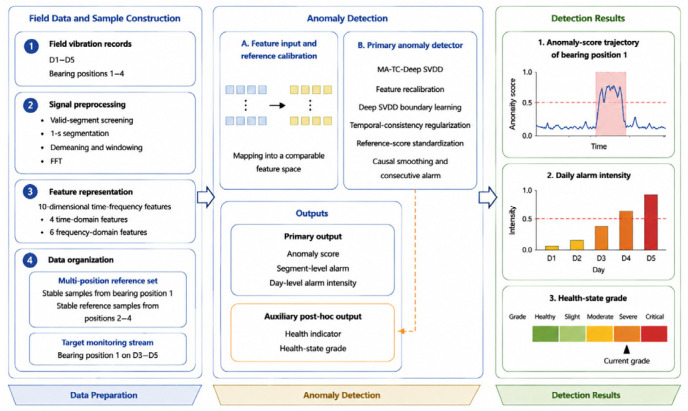
Overall workflow of the proposed MA-TC-Deep SVDD framework. The primary workflow produces segment-level anomaly scores and alarm decisions, while the auxiliary health-state module provides post hoc severity-oriented interpretation without affecting the anomaly detection threshold.

This partition keeps the training boundary, threshold selection, early-deviation analysis, main anomaly evaluation, and false-alarm verification logically separated. The overall workflow of the proposed MA-TC-Deep SVDD framework is illustrated in Figure 1.

### 2.2. Multi-Axle Reference Construction and Deep SVDD Boundary Learning

This subsection describes the core one-class boundary learning layer. Unlike prediction-error-based pipelines that first reconstruct or forecast vibration signals and then feed residual features into a separate classifier, MA-TC-Deep SVDD directly learns the compact distribution of stable monitoring segments in a latent feature space. The detector maps the standardized ten-dimensional feature vector into this latent space through a lightweight multilayer perceptron and uses the Deep SVDD compactness objective to describe the stable operating regime.

The key design is the construction of a multi-axle reference set. Stable samples from bearing position 1 provide target-position normal evidence, while stable samples from bearing positions 2–4 provide same-train operating-condition reference evidence. These reference positions are not assumed to be physically identical to the target position, because different monitored positions may have different installation conditions, vibration transmission paths, and load distributions. Therefore, positions 2–4 are used as auxiliary stable-control evidence rather than direct substitutes for the target bearing position.

The reference-guided boundary learning contains three operations. First, the standardized feature vector is mapped into a latent representation by the multilayer perceptron. Second, a lightweight feature-recalibration mechanism adjusts the contribution of different latent dimensions, allowing state-sensitive components to contribute more strongly to the distance score. Third, the normal hypersphere center is estimated from the multi-axle reference set, and reference samples are pulled toward this center through the compactness loss. In this way, the learned boundary is determined by stable multi-position evidence rather than by a single-position sample distribution.

**Figure 2:**
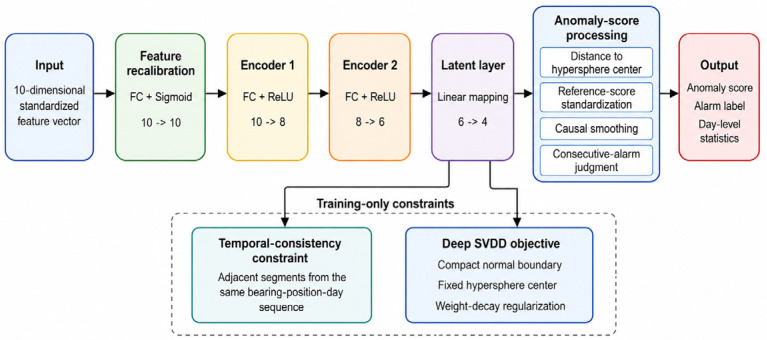
Architecture of the proposed MA-TC-Deep SVDD detector. The standardized input first undergoes feature recalibration and is subsequently mapped into a four-dimensional latent representation. Deep SVDD compactness and temporal-consistency constraints are applied during training, while reference-score standardization, causal smoothing, and consecutive-alarm judgment are used during inference.

The role of this detector is therefore different from ordinary Deep SVDD. Standard Deep SVDD mainly relies on normal samples from the target object, whereas the proposed design introduces same-train stable reference positions to make the learned boundary less sensitive to operating-condition fluctuation. The purpose of the multi-axle reference strategy is not to merge all monitored positions as the same object, but to provide additional same-train stable evidence for distinguishing localized target-position anomalous response from train-level operating-condition fluctuation. The architecture of the proposed MA-TC-Deep SVDD detector is illustrated in Figure 2.

The feature-mapping network consists of an input-side feature-recalibration layer and a compact, fully connected encoder. The standardized ten-dimensional input vector first passes through a fully connected sigmoid gating layer with dimensions 10–10. The recalibrated feature vector is subsequently mapped by two fully connected hidden layers with dimensions 10–8 and 8–6, respectively. A ReLU activation function is applied after each hidden layer. A final linear mapping layer with dimensions 6–4 produces the four-dimensional latent representation used for Deep SVDD boundary learning. The hypersphere center is initialized using the mean latent representation of the reference-training samples and remains fixed during network optimization. For the *l*-th hidden layer, the feature transformation is expressed as

(3) $$h_{i}^{\left(\ell \right)}=\phi \left(W^{\left(\ell \right)}h_{i}^{\left(\ell -1\right)}+b^{\left(\ell \right)}\right)$$

where *W*(*l*) and *b*(*l*) denote the weight matrix and bias vector of the *l*-th layer, and *φ*(·) is the nonlinear activation function. The latent representation of the input segment is obtained by

(4) $$z_{i}=g_{\theta }({\hat{x}}_{i})$$

where *g_θ_*(·) denotes the feature mapping network with trainable parameters *θ*. To enhance the contribution of informative latent dimensions, a lightweight feature recalibration operation is introduced as

(5) $$a_{i}=\sigma (W_{r}z_{i}+b_{r})$$

(6) $${\tilde{z}}_{i}=z_{i}⊙a_{i}$$

where *a_i_* is the recalibration weight vector, *σ*(·) is the sigmoid function, and ⊙ denotes element-wise multiplication.

The multi-axle reference set is composed of stable target-position samples and synchronous stable samples from other monitored positions. The center of the normal hypersphere is calculated as

(7) $$c=\frac{1}{N_{r}}\sum_{i\in R}{\tilde{z}}_{i}$$

where *R* denotes the reference-training set and *N_r_* is the number of reference samples. The compactness loss minimizes the distance between reference samples and the normal center:

(8) $$L_{comp}=\frac{1}{N_{r}}\sum_{i\in R}{∥{\tilde{z}}_{i}-c∥}_{2}^{2}$$

### 2.3. Temporal-Consistency Regularization and Engineering Alarm Decision

Field monitoring scores may contain isolated spikes caused by short-term shocks, local operating transitions, or segment-level noise. If each segment is judged independently, these spikes can easily be misinterpreted as alarms. To address this problem, temporal consistency is imposed within each bearing–position–day sequence so that adjacent monitoring segments maintain locally continuous latent responses:

(9) $$L_{tc}=\frac{1}{M}\sum_{i=1}^{M}{∥{\tilde{z}}_{i+1}-{\tilde{z}}_{i}∥}_{2}^{2}$$

The training objective combines three terms: compactness loss for normal-boundary learning, temporal-consistency loss for local sequence stability, and parameter regularization for controlling model complexity:

(10) $$L=L_{comp}+\lambda_{1}L_{tc}+\lambda_{2}\sum_{\ell }{∥W^{\left(\ell \right)}∥}_{F}^{2}$$

where *λ*_1_ and *λ*_2_ are balancing coefficients, and ‖·‖*F* denotes the Frobenius norm. The proposed detector was optimized using the Adam optimizer with an initial learning rate of $1\times 10^{-3}$, a batch size of 64, a maximum of 100 training epochs, and a weight-decay coefficient of $1\times 10^{-5}$. The temporal-consistency balancing coefficient was selected using only the reference-validation subset and was subsequently fixed for all evaluation subsets. The normal hypersphere center was initialized from the mean latent representation of the reference-training samples and was kept fixed during gradient-based optimization. No transition-evaluation, main-deviation-evaluation, or stable-control samples were used for model training or hyperparameter selection.

After training, the anomaly degree of each segment is measured by the squared distance between its recalibrated latent representation and the learned normal center:

(11) $$d_{i}={∥{\tilde{z}}_{i}-c∥}_{2}^{2}$$

The raw distance scores are then converted into an engineering alarm variable through three steps. First, the raw distance is standardized using the reference-score distribution, which makes scores from field segments comparable with the stable regime:

(12) $$s_{i}=max\left(0,\frac{d_{i}-\mu_{ref}}{\sigma_{ref}+\varepsilon }\right)$$

where *μ_ref_* and *σ_ref_* are the mean and standard deviation of reference scores. Second, a causal moving average is used to reduce isolated score spikes without using future information:

(13) $${\overset{¯}{s}}_{i}=\frac{1}{w}\sum_{k=0}^{w-1}s_{i-k}$$

where *w* is the smoothing window length. In the experiments, *w* is set to 5 according to the validation performance and engineering smoothing requirement. Third, a segment is reported as an alarm only when the smoothed score continuously exceeds the threshold for a predefined number of consecutive segments:

(14) $$y_{i}=I\left(\sum_{k=0}^{L_{c}-1}I({\overset{¯}{s}}_{i-k}>\tau )=L_{c}\right)$$

where *τ* is the alarm threshold, *L_c_* is the minimum number of consecutive exceedance segments, and I(·) denotes the indicator function. In the experiments, *τ* is determined as the 0.99 quantile of the reference-score distribution, and *L_c_* is set to 3. No transition-evaluation, main-deviation, or stable-control samples are used to determine this threshold. It should be noted that causal smoothing and consecutive-exceedance judgment are designed mainly for persistent state deviation. A larger smoothing window or consecutive length can reduce false alarms caused by isolated spikes, but may also suppress short-duration transient anomalies and increase alarm delay. Therefore, the selected setting represents a trade-off between false-alarm suppression and timely response. In this study, the method is positioned for maintenance-oriented monitoring of persistent abnormal responses rather than immediate detection of single impulsive events.

For engineering interpretation, segment-level alarms are further aggregated into day-level statistics. These statistics do not replace the segment-level detector; rather, they summarize whether the abnormal response is occasional or persistent within a monitoring day. The daily alarm intensity is defined as

(15) $$I_{d}=\frac{1}{n_{d}}\sum_{i\in d}y_{i}$$

where *d* denotes a monitoring day and *n_d_* is the number of valid segments on that day. In addition, the proportion of high-score segments can be calculated as

(16) $$P_{d}=\frac{1}{n_{d}}\sum_{i\in d}I({\overset{¯}{s}}_{i}>\tau )$$

The daily alarm intensity and high-score proportion jointly distinguish occasional high-score points from persistent daily abnormal responses. This design makes the detector more suitable for field maintenance interpretation, where continuous deviation is more meaningful than a single isolated exceedance.

### 2.4. Post Hoc Health-State Interpretation Based on APG-TA-SAE-DMHI

The main output of this study is the MA-TC-Deep SVDD anomaly detection result. This module is auxiliary and is not part of the primary anomaly detector. However, a binary alarm alone cannot fully describe how the target bearing position evolves across consecutive monitoring days. Therefore, a post hoc health-state interpretation module is retained after anomaly detection. Existing studies on health-index construction show that trend consistency and state-evolution description are useful when labels are weak and health-state boundaries are fuzzy [18,19,20]. Following this idea, an anomaly-prior-guided temporal-attention stacked autoencoder with a dynamic Mahalanobis health index, denoted as APG-TA-SAE-DMHI, is used to transform the detected deviation into a severity-oriented state description.

The APG-TA-SAE-DMHI module contains four operations. First, anomaly-prior weights are calculated from the anomaly scores so that samples closer to the stable regime have a higher influence when constructing the health reference. Second, a temporal-attention SAE encodes short-window state continuity and suppresses unstable local fluctuations in the health representation. Third, a dynamic Mahalanobis distance measures the deviation between each latent health feature and the weighted health reference. Fourth, the distance is normalized into a bounded health indicator and mapped to qualitative grades such as healthy, slight deviation, obvious deviation, and significant abnormality.

**Figure 3:**
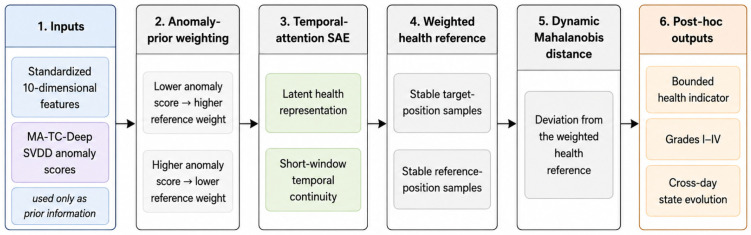
Auxiliary post hoc health-state interpretation module based on APG-TA-SAE-DMHI. The anomaly score is used only to construct prior weights for health-reference estimation. The resulting health indicator and state grades do not affect the primary anomaly threshold or binary alarm decision.

This module uses the same standardized time-frequency features as the anomaly detector, but it has no feedback path to the MA-TC-Deep SVDD alarm threshold. Its function is to explain severity evolution after anomaly detection rather than to redefine whether a segment is abnormal. Therefore, the binary anomaly decision, alarm threshold, and reported detection metrics are determined only by MA-TC-Deep SVDD. The structure and information flow of the auxiliary post hoc health-state interpretation module are illustrated in Figure 3.

Anomaly-prior weighting gives higher reference influence to samples with lower anomaly scores, thereby reducing the influence of suspicious segments on the health reference. The anomaly-prior weight is defined as

(17) $$\omega_{i}=exp(-\beta {\overset{¯}{s}}_{i})$$

where *β* controls the sensitivity of the weight to the anomaly score. A weighted health reference center is then calculated in the latent health space:

(18) $$\mu_{h}=\frac{\sum_{i\in R}\omega_{i}h_{i}}{\sum_{i\in R}\omega_{i}}$$

The deviation of each segment from the health reference is measured by the Mahalanobis distance:

(19) $$D_{i}=\sqrt{\left(h_{i}-\mu_{h}{)}^{T}\Sigma_{h}^{-1}(h_{i}-\mu_{h}\right)}$$

where Σ*_h_* is the covariance matrix of the reference health features. Finally, the health indicator is normalized into a bounded form:

(20) $$HI_{i}=exp(-D_{i})$$

A larger health indicator indicates a state closer to the health reference, whereas a smaller value indicates a stronger deviation from the stable regime. The grading result summarizes the detected cross-day evolution into four categories: healthy, slight deviation, obvious deviation, and significant abnormality. It should be regarded as a post hoc explanation layer rather than an additional decision layer.

### 2.5. Evaluation Protocol

The evaluation protocol follows the decision hierarchy of the proposed framework. The primary task is anomaly detection, so the main metrics include AUC, average precision (AP), precision, recall, F1-score, and false-alarm rate (FAR). Because the field data do not contain disassembly-confirmed fault labels, maintenance-confirmed fault types, or complete life-to-failure trajectories, the evaluation uses weak relative labels derived from cross-day feature changes and multi-position comparison. Therefore, the positive samples in this study should be interpreted as persistent state-deviation samples rather than confirmed bearing-fault samples. Segments from bearing position 1 on D4–D5 are treated as positive samples only for evaluating the ability to detect persistent anomalous responses, while reserved stable-control segments from positions 2–4 and stable reference-validation samples are treated as negative samples. The transitional samples on D3 are used mainly for early-deviation analysis and are not involved in threshold selection or hard-label performance statistics. All baseline methods use the same 10-dimensional feature input, the same sample partition, and the same validation-based threshold logic, so that the comparison focuses on the detector rather than on differences in data preprocessing. Specifically, OCSVM uses a radial basis function kernel with ν=0.05 and γ=0.1. Isolation Forest uses 200 estimators and sets the maximum number of samples for each estimator to $min(n_{train},256)$. The autoencoder baseline adopts a 10–8–4–8–10 architecture and is trained using Adam with a learning rate of $1\times 10^{-3}$, a batch size of 64, a maximum of 150 epochs, and an early-stopping patience of 15 epochs. Standard Deep SVDD uses a 10–8–6–4 architecture, a learning rate of $1\times 10^{-3}$, a weight-decay coefficient of $1\times 10^{-5}$, a batch size of 64, and a maximum of 100 epochs. All methods use the same standardized feature input and the same mutually separated data subsets.

The post hoc health-state module is evaluated only as supportive evidence. Monotonicity and trend consistency are used to check whether the health indicator follows the cross-day deviation process, while stable-segment fluctuation and multi-axle consistency are used to verify whether the interpretation remains stable on reference positions. These criteria assess the explanatory quality of APG-TA-SAE-DMHI and do not replace the anomaly detection metrics.

The field dataset originates from a railway engineering monitoring project. Owing to confidentiality restrictions, vehicle-identifying information, route details, detailed sensor specifications, mounting information, and service-run metadata cannot be publicly released. This restriction does not affect the reporting of the sampling frequency, segment length, frequency-domain processing, data-partition strategy, model architecture, training settings, or evaluation protocol used in this study.

## 3. Results and Discussion

The experiments are organized in the same order as the proposed pipeline. The cross-day anomaly evolution is first examined on the target bearing position. Then, cross-position evidence and benchmark results are used to verify localization and detection performance. Finally, the post hoc health-state module is discussed only as supportive severity evidence.

### 3.1. Cross-Day Anomaly Evolution of Bearing Position 1

**Figure 4:**
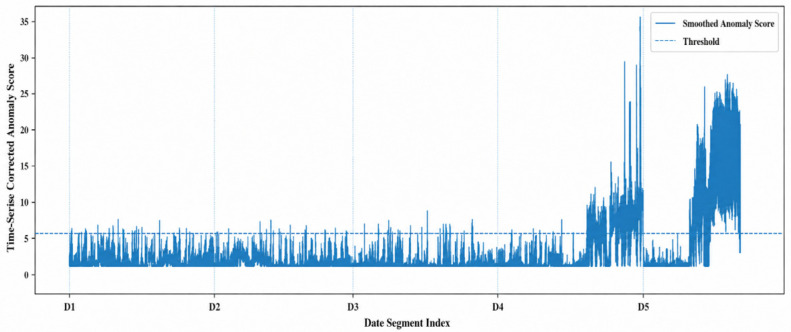
Segment-level anomaly-score trajectory of bearing position 1.

The experimental analysis first examines the target bearing position across consecutive days. Figure 4 shows the segment-level anomaly-score trajectory of bearing position 1. The scores remain low and comparatively stable on D1–D2, begin to fluctuate upward on D3, and increase persistently on D4–D5. This suggests that the observed response is not an isolated score spike, but a cross-day shift from a stable regime toward persistent state deviation under the current weak-label interpretation.

**Figure 5:**
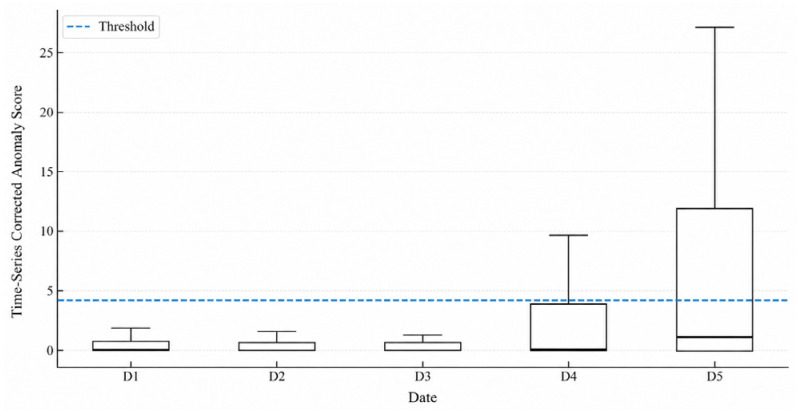
By-date distribution of anomaly scores for bearing position 1.

Figure 5 presents the by-date distribution of anomaly scores for bearing position 1. The daily medians and upper quartiles remain low on D1–D2, increase on D3, and rise markedly on D4–D5. The widening interquartile range on the later days indicates that high-score segments become more frequent and more dispersed during the daily monitoring period, which is consistent with a persistent anomalous response rather than an occasional disturbance.

### 3.2. Cross-Position Validation and Detection Benchmarking

**Figure 6:**
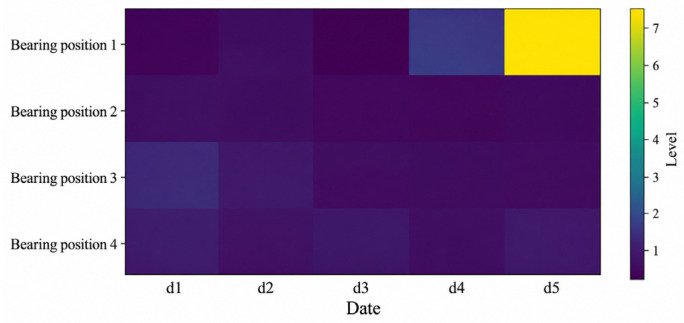
Multi-position day-level anomaly-intensity heatmap.

Figure 6 compares the day-level anomaly intensity across monitored positions. The strong rise is concentrated on bearing position 1, whereas bearing positions 2–4 remain at relatively low anomaly-score levels. This cross-position contrast weakens the explanation that the score increase is caused only by shared train-level operating-condition fluctuations and supports the judgment of a localized, bearing-position-specific deviation.

**Figure 7:**
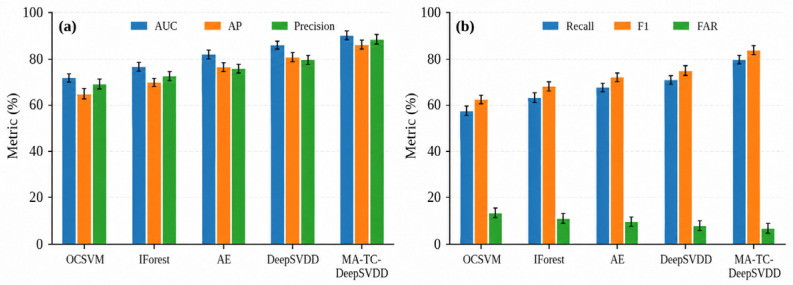
Performance comparison of different anomaly detection methods: (**a**) AUC, AP, and Precision; (**b**) Recall, F1-score, and FAR on stable-control samples.

The detection performance of different methods is summarized in Table 2 and Figure 7. Under the current weak-label evaluation protocol, MA-TC-Deep SVDD achieves higher average ranking ability and alarm reliability than the compared baselines. This improvement is not obtained simply by issuing more alarms, because the FAR on stable-control samples is reduced to 0.047.

**Figure 8:**
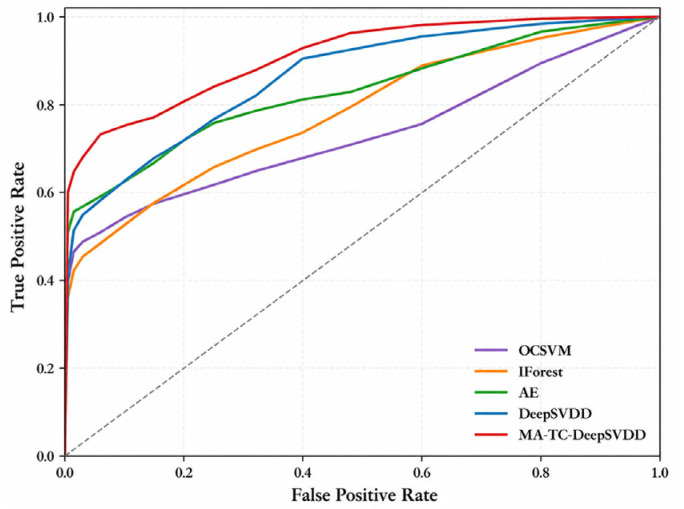
ROC curves of different anomaly detection methods on the field monitoring dataset. The gray dashed diagonal line represents the performance of a random classifier with no discrimination ability (AUC = 0.5).

Figure 8 further compares different methods through ROC curves. MA-TC-Deep SVDD shows a higher ROC profile than the compared baselines across most false-positive-rate ranges, indicating that the performance improvement under the current weak-label evaluation protocol is not limited to a single operating threshold.

The ablation results in Table 3 further clarify the contribution of each component under the current evaluation protocol. The temporal-consistency constraint improves the continuity of anomaly responses, leading to higher recall and F1-score than standard Deep SVDD. The multi-axle reference mechanism further reduces FAR by introducing same-train stable samples from other monitored bearing positions. The complete MA-TC-Deep SVDD model obtains the highest average performance among the ablation variants, suggesting that the two components provide complementary effects for persistent state-deviation detection.

### 3.3. Auxiliary Post Hoc Health-State Interpretation

**Figure 9:**
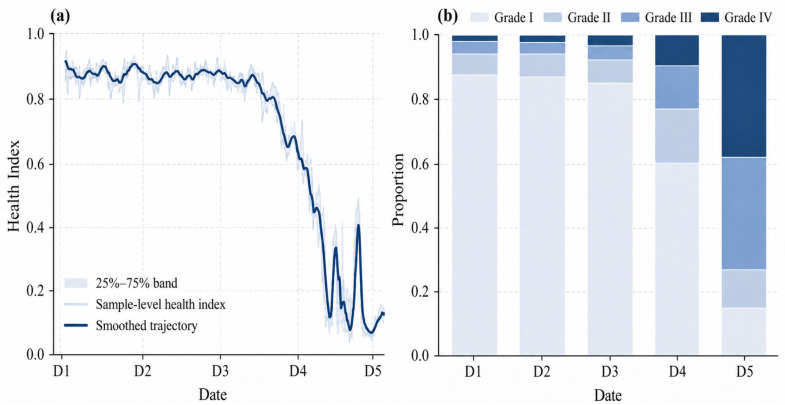
Post hoc health-state interpretation of bearing position 1: (**a**) health-indicator trajectory; (**b**) daily health-state composition.

Although the primary objective of this study is anomaly detection, the APG-TA-SAE-DMHI module provides an auxiliary, severity-oriented view of the detected deviation. Figure 9 shows that the health indicator remains high on D1–D2, begins to decline on D3, and drops substantially on D4–D5. The daily state composition exhibits a consistent pattern: D1–D2 is dominated by Grades I–II, D3 shows an expansion of Grades II–III, and D4–D5 is dominated by Grades III–IV.

This post hoc health-state view is consistent with the anomaly detection results and converts the detected cross-day deviation into a smoother engineering summary. However, it is not used to determine the anomaly threshold, the binary alarm result, or the detection metrics reported in Table 2.

As shown in Table 4, the auxiliary modules improve the trend description of the health indicator. TA-SAE-DMHI obtains the highest monotonicity, while APG-TA-SAE-DMHI achieves the best trend consistency, the lowest stable fluctuation, and the highest multi-axle consistency. Therefore, the complete auxiliary module provides a more balanced severity-oriented interpretation. However, these results should be interpreted only as supportive post hoc evidence and are not used for primary anomaly decision-making.

### 3.4. Engineering Discussion

The field case indicates that anomaly detection should serve as the primary operational layer in weak-label monitoring scenarios. In the present dataset, the anomaly-score sequence provides the main evidence that bearing position 1 remains stable on D1–D2, begins to deviate on D3, and exhibits persistent state deviation on D4–D5. In contrast, bearing positions 2–4 remain at relatively low anomaly levels, suggesting that the detected response is more likely to be position-specific than a train-level operating fluctuation. From an engineering perspective, the proposed method is therefore positioned as a deployable anomaly-warning framework, while the health indicator is used only to explain severity after the warning signal has emerged.

This study still has practical limitations. The field case does not include exhaustive fault-type labels, disassembly-confirmed fault records, or a complete life-to-failure trajectory. Therefore, the framework is positioned as anomaly detection with post hoc health-state interpretation rather than full fault-type diagnosis or prognostics. In addition, because temporal smoothing and consecutive-alarm judgment are used, the current framework is more suitable for persistent state deviation than for instantaneous impact-type anomalies. Sudden bearing damage with very short duration may require a shorter smoothing window, a smaller consecutive-alarm length, or a parallel impact-sensitive detector. Future work should examine cross-train adaptation, online reference updating, and joint use of vibration, speed, current, and temperature signals.

## 4. Conclusions

This study proposed MA-TC-Deep SVDD, a field vibration-based anomaly detection framework for EMU traction motor bearings under weak-label and variable-condition monitoring scenarios. The framework combines compact time-frequency representation, multi-axle reference-guided Deep SVDD boundary learning, temporal consistency, reference-score processing, and consecutive alarm judgment.

The field case over D1–D5 showed that bearing position 1 remained stable on D1–D2, exhibited transitional deviation on D3, and developed persistent state deviation on D4–D5, while the other monitored bearing positions remained comparatively stable. This cross-position contrast supports the interpretation that the detected deviation is localized rather than a general train-level fluctuation.

Under the current weak-label evaluation protocol, MA-TC-Deep SVDD achieved higher average anomaly detection performance than the compared baseline methods, with AUC = 0.909, AP = 0.872, Precision = 0.887, Recall = 0.802, F1 = 0.843, and FAR = 0.047. The ablation results further suggest that the multi-axle reference strategy and temporal-consistency constraint contribute to reducing false alarms and improving persistent-deviation detection. The APG-TA-SAE-DMHI module provides an auxiliary severity-oriented description of the detected cross-day deviation without changing the anomaly detection-centered decision logic.

The current framework should be interpreted as a weak-label field anomaly-warning and post hoc severity-interpretation method. It cannot replace disassembly-confirmed fault-type diagnosis or remaining useful life prediction.

Future work will focus on cross-train adaptation, online reference updating, and multi-source monitoring fusion using vibration, speed, current, and temperature signals. If richer fault labels and complete life-to-failure trajectories become available, the framework can be further extended from anomaly warning to more detailed fault-type recognition and prognostic analysis.

## Figures and Tables

**Table 1:** Sample partition used in the field case study.

Subset	Source	Role	Samples
Reference train	Bearing position 1 on D1–D2 + screened stable fragments from bearing position 1 on D3 + stable segments from positions 2–4	Learn normal boundary	178,234
Reference validation	Reserved stable segments from the same reference pool	Tune hyperparameters and alarm threshold	76,392
Transition evaluation	Remaining bearing position 1 samples on D3 after stable-fragment screening	Early-deviation analysis; not used for threshold selection or hard-label metrics	32,582
Main deviation evaluation	Bearing position 1 on D4–D5	Primary anomaly evaluation	54,940
Stable control	Reserved stable segments from positions 2–4, disjoint from the multi-axle reference pool	Independent false-alarm verification	262,417

**Table 2:** Performance comparison of different anomaly detection methods.

Method	AUC	AP	Precision	Recall	F1	FAR
OCSVM	0.731 ± 0.000	0.648 ± 0.000	0.687 ± 0.000	0.582 ± 0.000	0.630 ± 0.000	0.124 ± 0.000
IForest	0.781 ± 0.012	0.709 ± 0.015	0.738 ± 0.008	0.644 ± 0.013	0.688 ± 0.011	0.107 ± 0.007
AE	0.828 ± 0.014	0.776 ± 0.009	0.776 ± 0.013	0.689 ± 0.010	0.730 ± 0.012	0.093 ± 0.006
Deep SVDD	0.862 ± 0.010	0.814 ± 0.012	0.804 ± 0.009	0.716 ± 0.014	0.757 ± 0.011	0.082 ± 0.005
MA-TC-Deep SVDD	0.909 ± 0.007	0.872 ± 0.010	0.887 ± 0.006	0.802 ± 0.011	0.843 ± 0.008	0.047 ± 0.004

**Table 3:** Ablation study of the proposed detector.

Method	AUC	AP	Precision	Recall	F1	FAR
Deep SVDD	0.862 ± 0.010	0.814 ± 0.012	0.803 ± 0.009	0.716 ± 0.014	0.757 ± 0.011	0.082 ± 0.005
TC-Deep SVDD	0.881 ± 0.013	0.838 ± 0.008	0.827 ± 0.010	0.742 ± 0.015	0.782 ± 0.012	0.073 ± 0.007
MA-Deep SVDD	0.889 ± 0.006	0.849 ± 0.011	0.856 ± 0.007	0.760 ± 0.009	0.805 ± 0.010	0.059 ± 0.004
MA-TC-Deep SVDD	0.909 ± 0.007	0.872 ± 0.010	0.887 ± 0.006	0.802 ± 0.011	0.843 ± 0.008	0.047 ± 0.004

**Table 4:** Auxiliary evaluation of post hoc health-state interpretation methods.

Auxiliary Method	Monotonicity	Trend Consistency	Stable Fluctuation	Multi-Axle Consistency
SAE-DMHI	0.698	0.658	0.216	0.296
APG-SAE-DMHI	0.787	0.758	0.210	0.335
TA-SAE-DMHI	0.814	0.789	0.205	0.344
APG-TA-SAE-DMHI	0.809	0.798	0.203	0.347

## Data Availability

The data supporting this study are available from the corresponding author upon reasonable request and subject to approval by the data owner. Public release of the raw vibration records and associated acquisition metadata is restricted because the dataset contains confidential technical information related to high-speed EMU systems. Vehicle-identifying information, route details, detailed sensor specifications, mounting configuration, and service-run metadata cannot be publicly disclosed. Derived feature data or selected non-identifying information may be shared for academic collaboration where permitted by the relevant data-use requirements.
